# Association between oral intake magnesium and sarcopenia: a cross-sectional study

**DOI:** 10.1186/s12877-022-03522-5

**Published:** 2022-10-22

**Authors:** Shih-Wei Yang, Yuan-Yuei Chen, Wei-Liang Chen

**Affiliations:** 1 Division of Plastic and Reconstruction Surgery, Department of Surgery, Tri-service General Hospital, National Defense Medical Center, Taipei, Taiwan, Republic of China; 2 Division of Family Medicine, Department of Family and Community Medicine, Tri-Service General Hospital; and School of Medicine, National Defense Medical Center, Taipei, Taiwan, Republic of China; 3 Department of General Medicine, Tri-Service General Hospital; and School of Medicine, National Defense Medical Center, Taipei, Taiwan, Republic of China; 4 Division of Geriatric Medicine, Department of Family and Community Medicine, Tri-Service General Hospital; and School of Medicine, National Defense Medical Center, Number 325, Section 2, Chang-gong Rd, Nei-Hu District, 114 Taipei, Taiwan; 5 Department of Pathology, Tri-Service General Hospital Songshan Branch; and School of Medicine, National Defense Medical Center, Taipei, Taiwan, Republic of China

**Keywords:** Magnesium, Sarcopenia

## Abstract

**Background:**

Sarcopenia is a common skeletal muscle disorder in the elderly population. The patients with sarcopenia increased the cost of care and the risk for hospitalization. Magnesium deficiency might increase reactive oxygen species and protein damage. The purpose of our study was to demonstrate the relation between oral intake magnesium and sarcopenia by European Working Group on Sarcopenia in Older People (EWGSOP) 2 definition.

**Methods:**

Our study included 2532 participants with 1310 males and 1222 females. The multiple logistic regression model was designed to test the cross-sectional protective outcome of oral intake magnesium for sarcopenia.

**Results:**

Oral intake magnesium had a protective outcome with sarcopenia (odd ratio (OR) = 0.997, 95% CI = 0.996, 0.998, *P* < 0.001). After fully adjusted, the significance persisted with OR = 0.998 (95% CI = 0.996, 0.999, *P* < 0.001).

**Conclusion:**

Results of the present study showed the dose dependent relationship between oral intake magnesium and sarcopenia. Sufficient oral intake magnesium might prevent patient from sarcopenia.

**Supplementary Information:**

The online version contains supplementary material available at 10.1186/s12877-022-03522-5.

## Introduction

Sarcopenia, one of the primary diseases of the elderly population, is a common skeletal muscle disorder that involves the progressive loss of skeletal muscle function and mass [1]. To date, the most widely cited diagnostic criteria is that proposed by the European Working Group on Sarcopenia in Older People (EWGSOP) and updated as EWGSOP2 in 2019 [2]. According to EWGSOP2, muscle strength, muscle quantity and physical performance were measured to evaluate sarcopenia [2]. Loss of skeletal muscle mass and function causes the risk of falls, fractures, physical disability, use of hospital services, poor quality of life, and death [3]. The patients with sarcopenia increase the risk for hospitalization and the cost of care during hospitalization [4]. In 2000, there was approximately $18.5 billion ($10.8 billion in men, $7.7 billion in women) health-care cost in the America due to sarcopenia [5]. Malnutrition plays a key role in the pathogenesis of sarcopenia [6] [7] [8]. There was a growing evidence base linking nutrition with greater muscle strength and better physical performance outcomes in patient with sarcopenia [9]. However, these articles paid more attention on protein [10], long-chain polyunsaturated fatty acids [11], vitamin D [12], and antioxidant nutrients [13]. Best to our knowledge, no previous data had discussed oral intake electrolyte and sarcopenia.

Magnesium is the second most intracellular cation after potassium in human. Several metabolic pathways and fundamental cellular activities including peptide and DNA synthesis, signal transduction, blood glucose control, stimulus-contraction coupling, stimulus-secretion coupling, and ion channel translocation related to magnesium [14]. Total body magnesium content is mainly located in bone and soft tissue [15]. The recommended daily intake of magnesium is 320 and 420 mg for adult women and men, respectively. Absorption mainly (80–90%) occurs in the jejunum and colon through passive and active transport [16]. Reabsorption of magnesium mainly occurred in the thin ascending limb of Henle (70%) and partially occurred in the proximal tubule and distal collecting tubule. Hypomagnesemia related to several clinical manifestations including neuromuscular and cardiovascular manifestations. Muscle spasms, muscle cramps, tremors, atrial fibrillation and ventricular arrhythmias were the specific symptoms of hypomagnesemia [17]. Further, electrolyte and hormone imbalanced including hypocalcemia, hypokalemia, and hypoparathyroidism were also associated with hypomagnesemia.

According to a previous study, magnesium deficiency might increase reactive oxygen species and lipid and protein damage and associate with damage of muscle [18]. The purpose of our study was to demonstrate the relation between oral intake magnesium and sarcopenia by EWGSOP2 definition.

## Materials and methods

### Study design and participants

**Fig. 1:**
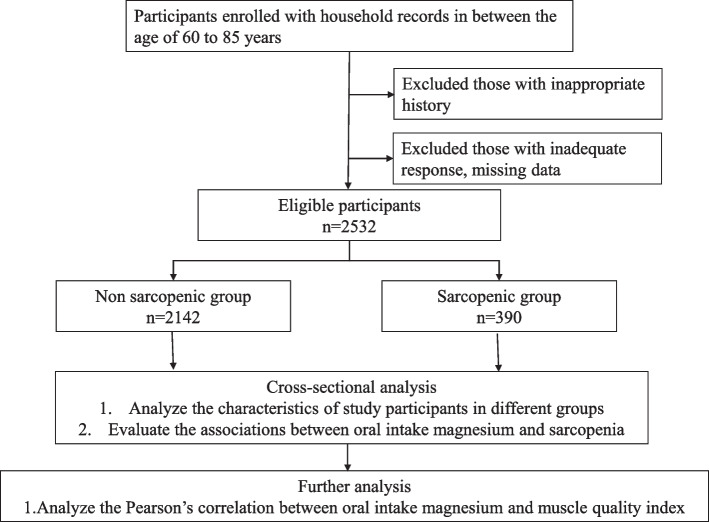
Flow chart representing the steps of analysis performed in the study

We conducted a cross-sectional study using the National Health and Nutrition Examination Survey (NHANES) database in 1999-2002 period. The data included overall home interviews and laboratory assessments conducted by the National Center for Health Statistics (NCHS). Institutional Review Board (IRB) approval and documented consent was obtained from participants. The exclusion criteria were dysphagia; history of gastrointestinal tract disease; inflammatory bowel disease; history of cancer; participation in resistance training; participation regularly using steroids, diuretics, hormones, and growth factors; and subjects without complete clinical data, laboratory results, or medical history. (Fig. 1).

### EWGSOP2 guideline

The EWGSOP published a sarcopenia definition in 2010 [19]. In early 2018, the Working Group met again (EWGSOP2) to update the definition of sarcopenia. In the EWGSOP2, low muscle strength as the primary parameter of sarcopenia. A sarcopenia diagnosis is confirmed by low muscle strength with the presence of low muscle quantity or quality. Severe sarcopenia was considered with three components, including low muscle strength, low muscle quantity/quality and low physical performance.

### Measurement: muscle quality index

The NHANES documentation provided measurement of the muscle quality index. We listed these methods and cut points to estimate components of muscle quality index in Table-S1.

### Measurement of oral intake magnesium

Total magnesium intake in milligrams per day calculated from dietary information with 24 hours dietary recalls. Subjects were required to recall all intakes in one day period prior before the questionnaire. A measuring guides and a food model booklet was available for participants to help in reporting food amounts.

### Assessment of Covariables

A computer-aided individual interviewing method was used for the collection of participants’ information. Demographic information, including sex, age, race was assembled. Smoking and drinking status were assessed by a detailed survey. We also recorded self-reported comorbidities, including cancer and cardiovascular disease. Based on the guidelines of Centers for Disease Control and Prevention, biochemical analyses in the database used standard methods.

### Statistical analysis

SPSS (Version 18.0 for Windows, SPSS, Inc., Chicago, IL) was used for all the statistical analyses. Descriptive information of continuous and categorical covariates is presented as the mean (standard deviation) and several observations with percentage (%). Continuous variables were used Student’s *t* tests and categorical variables were used the ***χ*** ^2^ test for the comparison of characteristics and covariates across subgroups. The odds ratio (OR) with 95% confidence interval (CI) for sarcopenia was estimated by multivariate logistic regression analysis. We analyzed the association between oral intake magnesium and muscle quality index using a linear regression model. The beta coefficient is the degree of change in the muscle quality index for every 1-unit of change in the oral intake magnesium. The 2-sided *P* values less than 0.05 indicated significant differences. Four extended models were used for covariate adjustments. Model 1 examined unadjusted odd ratio (ORs). Model 2 presented ORs after adjusting sex, age, and race. Model 3 was adjusted for the variables included in Model 2 + the body mass index (BMI), Albumin, total calcium, creatinine, fasting glucose, C-reactive protein (CRP), daily carbohydrate intake, daily total fat intake, and daily protein intake. The variables in model 3 + the smoking history, comorbidities and education level were adjusted for model 4.

## Result

### Characteristics of the subjects

2532 subjects with 1310 males and 1222 females were included in our study. We separated subjects into two groups based on the EWGSOP definition of sarcopenia. (Table 1) The mean age were 73.14 ± 9.59 years in the sarcopenic group and 63.90 ± 9.32 years in the non-sarcopenic group, respectively. It was also observed that the mean oral intake magnesium amount was higher for non-sarcopenic group at 276.06 ± 142.04 compared to 235.97 ± 112.61 in the sarcopenic group (*P* < 0.001).

**Table 1:** Characteristics of study participants

Characteristics of study participants	Sarcopenia	Non-sarcopenia	***P*** value
	(***n*** = 390)	(***n*** = 2142)	
**Age at screening**	73.14 (9.59)	63.90 (9.32)	0.227
**Body mass index (kg/m**^**2**^**)**	27.89 (4.70)	28.79 (5.58)	0.421
**Creatinine (mg/dL)**	0.92 (0.67)	0.88 (0.50)	0.001
**Glucose (mg/dL)**	107.81 (43.25)	102.39 (36.39)	0.001
**C-reactive protein (mg/dL)**	0.57 (0.98)	0.46 (0.75)	0.008
**Albumin(g/dL)**	4.22 (0.31)	4.31 (0.28)	0.211
**Total Calcium (mg/dL)**	9.47 (0.44)	9.43 (0.40)	0.178
**Magnesium (mg/dL)**	235.97 (112.61)	276.06 (142.04)	< 0.001
**Carbohydrate (gm)**	210.63 (94.13)	239.32 (114.20)	0.002
**Total fat (gm)**	61.79 (31.69)	72.23 (43.07)	< 0.001
**Protein (gm)**	64.26 (28.22)	74.22 (37.50)	< 0.001
**Dietary fiber (gm)**	14.16 (8.09)	16.78 (10.76)	< 0.001
**Comorbidity**	0.92 (0.99)	0.60 (0.89)	0883
**Gender (male)**	34.1%	54.9%	< 0.001
**Race/ethnicity**			
**Mexican American**	15.1%	19.2%	0.280
**Other Hispanic**	4.4%	4.2%	
**Non-Hispanic White**	63.1%	59.8%	
**Non-Hispanic Black**	14.1%	14.6%	
**Smoking**	52.1%	43.5%	0.008
**Drinking**	53.5%	67.6%	< 0.001
**Education level past high school**	57.8%	65.8%	0.003
**Congestive heart failure**	7.4%	3.2%	< 0.001
**Coronary heart disease**	9.2%	6.8%	0.109
**Angina**	6.9%	5.6%	0.291
**Heart attack**	9.0%	5.7%	0.016
**Stroke**	0.5%	0.7%	1.000
**Emphysema**	6.2%	2.4%	< 0.001

### Association between oral intake magnesium and sarcopenia

In Table 2, we showed the association between different oral intake elements and sarcopenia. After adjusted all covariates, the association only showed in oral intake magnesium and potassium.

**Table 2:** The association between different oral intake elements and sarcopenia

Components of sarcopenia								
Oral intake elements	Model 1	***P*** Value	Model 2	***P*** Value	Model 3	***P*** Value	Model 4	***P*** Value
**magnesium (mg)**	0.997 (0.996-0.998)	< 0.001	0.999 (0.998-1.000)	0.028	0.997 (0.996-0.999)	0.003	0.998 (0.996-0.999)	0.007
**Calcium (mg)**	1.000 (0.999-1.000)	0.047	1.000 (1.000-1.000)	0.835	1.000 (1.000-1.000)	0.988	1.000 (1.000-1.000)	0.883
**Phosphorate (mg)**	1.000 (0.999-1.000)	< 0.001	1.000 (1.000-1.000)	0.491	1.000 (0.999-1.000)	0.357	1.000 (0.999-1.000)	0.442
**Iron (mg)**	0.981 (0.968-0.996)	0.010	0.993 (0.979-1.008)	0.360	0.992 (0.973-1.011)	0.415	0.994 (0.975-1.014)	0.567
**Zinc (mg)**	0.966 (0.948-0.985)	< 0.001	0.992 (0.975-1.009)	0.335	0.986 (0.963-1.010)	0.262	0.988 (0.966-1.011)	0.305
**Copper (mg)**	0.925 (0.804-1.064)	0.276	1.042 (0.966-1.125)	0.288	1.040 (0.962-1.124)	0.326	1.046 (0.968-1.131)	0.255
**Sodium (mg)**	1.000 (1.000-1.000)	< 0.001	1.000 (1.000-1.000)	0.871	1.000 (1.000-1.000)	0.533	1.000 (1.000-1.000)	0.508
**Potassium (mg)**	1.000 (1.000-1.000)	< 0.001	1.000 (1.000-1.000)	0.047	1.000 (1.000-1.000)	0.017	1.000 (1.000-1.000)	0.034

*BMI* Body mass index, *CRP* C-reactive protein

Adjusted covariates:

Model 1 = Unadjusted

Model 2 = Adjusted age, sex, race/ethnicity

Model 3 = Model 2 + BMI, Albumin, total calcium, creatinine, fasting glucose, CRP, daily carbohydrate intake, daily total fat intake, and daily protein intake

Model 4 = Model 3 + drinking history, smoking history, comorbidities, and education level

In Table 3, we performed logistic regression to exam the association between oral intake magnesium and sarcopenia. In Model 1, oral intake magnesium was significantly associated with sarcopenia (OR = 0.997, 95% confidence interval (CI) = 0.996, 0.998, *P* < 0.001) The significance persisted after adjusted all covariates in Model 4 (OR = 0.998, 95% CI = 0.996, 0.999, *P* = 0.007).

**Table 3:** Result of logistic regression predicting the presence of sarcopenia by oral intake magnesium

Components of sarcopenia								
	Model 1	***P*** Value	Model 2	***P*** Value	Model 3	***P*** Value	Model 4	***P*** Value
**Oral intake magnesium (mg)**	0.997 (0.996-0.998)	< 0.001	0.999 (0.998-1.000)	0.028	0.997 (0.996-0.999)	0.003	0.998 (0.996-0.999)	0.007

*BMI* Body mass index, *CRP* C-reactive protein

Adjusted covariates:

Model 1 = Unadjusted

Model 2 = Adjusted age, sex, race/ethnicity

Model 3 = Model 2 + BMI, Albumin, total calcium, creatinine, fasting glucose, CRP, daily carbohydrate intake, daily total fat intake, and daily protein intake

Model 4 = Model 3 + drinking history, smoking history, comorbidities, and education level

### Sensitivity analysis

Logistic regression was used for sensitivity analysis to compare the sarcopenic and non-sarcopenic groups and quartiles of oral intake magnesium. Similar outcomes were found for the relation between oral intake magnesium and sarcopenia in Table 4. In the unadjusted model, the highest quartile of oral intake magnesium had an odds ratio of 0.400 to be associated with sarcopenia (95% CI = 0.282, 0.568, *P* < 0.001). The odds ratio of subjects with the highest quartile of oral intake magnesium would have sarcopenia was 0.468 (0.274, 0.799) times fewer than subjects with the lowest quartile of oral intake magnesium after fully adjusted.

**Table 4:** Association between quartiles of oral intake magnesium and sarcopenia

		Sarcopenia	
**Models**	**Quartiles of oral intake magnesium**	**OR (95% CI)**	***P*** **Value**
**Model 1**	**Q2 v.s. Q1**	**0.937 (0.701-1.251)**	**0.658**
	**Q3 v.s. Q1**	**0.767 (0.568-1.036)**	**0.084**
	**Q4 v.s. Q1**	**0.400 (0.282-0.568)**	**< 0.001**
**Model 2**	**Q2 v.s. Q1**	**0.870 (0.635-1.192)**	**0.387**
	**Q3 v.s. Q1**	**0.871 (0.628-1.208)**	**0.409**
	**Q4 v.s. Q1**	**0.615 (0.421-0.900)**	**0.012**
**Model 3**	**Q2 v.s. Q1**	**0.782 (0.555-1.102)**	**0.160**
	**Q3 v.s. Q1**	**0.767 (0.518-1.134)**	**0.183**
	**Q4 v.s. Q1**	**0.453 (0.267-0.767)**	**0.003**
**Model 4**	**Q2 v.s. Q1**	**0.775 (0.548-1.097)**	**0.150**
	**Q3 v.s. Q1**	**0.794 (0.534-1.181)**	**0.255**
	**Q4 v.s. Q1**	**0.468 (0.274-0.799)**	**0.005**

*BMI* Body mass index, *CRP* C-reactive protein, *CI* Confidence interval, *OR* Odds ratio, *Q* Quartile

Adjusted covariates:

Model 1 = Unadjusted

Model 2 = Adjusted age, sex, race/ethnicity

Model 3 = Model 2 + BMI, Albumin, total calcium, creatinine, fasting glucose, CRP, daily carbohydrate intake, daily total fat intake, and daily protein intake

Model 4 = Model 3 + drinking history, smoking history, comorbidities, and education level

### Correlation between oral intake magnesium and muscle quality index

In Table 5, the coefficients of oral intake magnesium and muscle strength, SMI, and gait speed were 0.207, 0.134, and − 0.137. The linear regression analysis of the relation of oral intake magnesium and muscle quality index was showed in Table 6. The association of oral intake magnesium and muscle strength (β = 0.145, 95% CI = 0.118, 0.172), SMI (β = 0.001, 95% CI = 0.001, 0.002), and gait speed (β = − 0.000365, 95% CI = − 0.000471, − 0.000258) had statistically significant result (*P* < 0.001). After fully adjusted, the association of oral intake magnesium and muscle strength (β = 0.048, 95% CI = 0.012, 0.083), SMI (β = − 0.000149, 95% CI = − 000434, 0.000136), and gait speed (β = − 0.000171, 95% CI = − 0.000338, − 0.000005).

**Table 5:** Pearson correlation coefficient between oral intake magnesium and muscle quality index

Components of sarcopenia						
	Average peak force (Newtons)	Skeletal muscle index			Gait speed	
	**Correlation**	***P*** **Value**	**Correlation**	***P*** **Value**	**Correlation**	***P*** **Value**
**Oral intake magnesium (mg)**	0.207	< 0.001	0.134	< 0.001	− 0.137	< 0.001

**Table 6:** Association between oral intake magnesium and muscle quality index

Muscle quality index						
	Average peak force (Newtons)		Skeletal muscle index		Low gait speed	
**Models**	**OR (95% CI)**	***P*** **Value**	**OR (95% CI)**	***P*** **Value**	**OR (95% CI)**	***P*** **Value**
**Model 1**	0.145 (0.118-0.172)	< 0.001	0.001 (0.001-0.002)	< 0.001	− 0.000365 (− 0.000471- − 0.000258)	< 0.001
**Model 2**	0.043 (0.020-0.066)	< 0.001	−0.000356 (− 0.001- − 0.000032)	0.031	− 0.000253 (− 0.000359- − 0.000147)	< 0.001
**Model 3**	0.060 (0.025-0.096)	0.001	−0.000188 (− 0.000472-0.000096)	0.195	−0.000264 (− 0.000433- − 0.000096)	0.002
**Model 4**	0.048 (0.012-0.083)	0.009	−0.000149 (− 000434-0.000136)	0.306	−0.000171 (− 0.000338- − 0.000005)	0.044

*BMI* Body mass index, *CRP* C-reactive protein, *CI* Confidence interval, *OR* Odds ratio

Adjusted covariates:

Model 1 = Unadjusted

Model 2 = Adjusted age, sex, race/ethnicity

Model 3 = Model 2 + BMI, Albumin, total calcium, creatinine, fasting glucose, CRP, daily carbohydrate intake, daily total fat intake, and daily protein intake

Model 4 = Model 3 + drinking history, smoking history, comorbidities, and education level

## Discussion

In our presented study, the relationship between oral intake magnesium and the components of sarcopenia was closely inspected in 2532 adult population. Our results demonstrated strong relationships between oral intake magnesium and sarcopenia. Otherwise, based on the EWGSOP2, three components of sarcopenia all presented correlation with oral intake magnesium. To date, our study was the first article investigating the relation of oral intake magnesium and sarcopenia in a representative sample of the United States adult population.

Due to complicated pathogenesis, the development of sarcopenia remained controversial in the medical field. Previous study had mentioned different factors which associated with sarcopenia [20]. One of the main factors induced sarcopenia was the mitochondrial dysfunction [21]. Mitochondrial oxidative stress and dysfunction could be induced by intracellular magnesium deficiency [22]. Downregulating the electron transport chain increased the production of reactive oxygen species [23]. Low level of intracellular magnesium also suppressed the antioxidant defense system and reduced proteins such as superoxide dismutase, catalase, and glutathione [24]. Intracellular magnesium sufficient, in contrast, was found to suppress mitochondrial reactive oxygen species and improve mitochondrial function [25].

Intracellular and extracellular concentration of magnesium had thought to impact the synthesis of DNA and protein [26]. Magnesium regulated the onset of protein synthesis in activated frog oocytes and its rate in lymphocytes. Magnesium determines the onset of protein synthesis through the phosphatidylinositol-3-OH kinase (PI-3-K) pathway at mechanistic target of rapamycin (mTOR) phosphorylation of two translation-regulating proteins [27, 28]. One study measured intracellular concentration of magnesium and the rate of protein and DNA synthesis [29]. At the same magnesium concentration, the rate of DNA synthesis peaked as protein synthesis. This article provided strong evidence of magnesium as a regulator of DNA and protein synthesis.

Low magnesium status is also reported to associate with increasing reactive oxygen species and inflammation. Several articles had discussed that higher magnesium consumption was related to lower serum CRP [30]. People who takes magnesium at least 50 mg/day were 22% less likely to have higher level of plasma CRP compared with those dietary magnesium taking less than 50% of the recommended dietary allowance [31]. Magnesium supplement is associated with a lower serum CRP in these studies. One linear regression analysis of 3173 females aged 50–79 years found that magnesium supplement was associated with lower serum inflammatory biomarkers, such as CRP, interlukin-6 (IL-6), and tumor necrosis factor-α receptor 2 (TNF-α-R2, [32]].

Our study has several strengths. We screened the sarcopenia clearly by using EWGSOP2 guideline. We also screened other oral intake elements initially in our study. A large adult sample was included to discuss the protective outcome of oral intake magnesium for sarcopenia. In the current study, sufficient oral intake magnesium could reduce the incidence of sarcopenia. This result is not only effective, but also economical for preventing from sarcopenia in the public health system.

However, some limitations in our study should be identified. First, we used a cross-sectional database in which the oral intake magnesium was determined at single measure instead of a long observational period. Then, some information such as participants’ education levels and medical histories were based on questionnaires. The recall bias and other unknown bias might distort our results. Third, total magnesium intake was calculated from 24 hours dietary recalls and measured by guides and a food model booklet. This method caused inaccuracy of daily oral intake magnesium. Lastly, the factors of drug consumption, participants’ cognitive and nutritional status were not included our results. Thus, the confounding bias could not be estimated.

## Conclusion

In our presented study, we identified the protective outcome of oral intake magnesium with sarcopenia. The significance especially showed in muscle strength and gait speed. Otherwise, dose dependent relationship was observed between oral intake magnesium and sarcopenia. Our findings suggested that sufficient oral intake magnesium might prevent patient from sarcopenia. Further prospective studies may be warranted to find the curative effect of oral intake magnesium in the patient with sarcopenia.

## Supplementary Information

**Additional file 1 Supplement** **Table** **1.** Standard measurement protocol of muscle quality index in NHANES.

## Data Availability

The datasets used and/or analyzed during the current study are available from the NHANES database, https://www.cdc.gov/nchs/nhanes/index.htm.

## Abbreviations

EWGSOP: European Working Group on Sarcopenia in Older People
NHANES: National Health and Nutritional Examination Survey
NCHS: National Center for Health Statistics
DXA: Dual-energy X-ray absorptiometry
SMI: Skeletal muscle index
BMI: Body mass index
CRP: C-Reactive protein
OR: Odds ratio
CI: Confidence interval
ROC: Receiver operating characteristic curve
AUROC: Area under receiver operating characteristic curve
IL-6: Interlukin-6
TNF-α-R2: Tumor necrosis factor-α receptor 2
