# Supplementary material for: Association between oral intake magnesium and sarcopenia: a cross-sectional study
Source: BMC Geriatr. 2022 Oct 22;22:816. doi: 10.1186/s12877-022-03522-5 (PMC9587540; doi:10.1186/s12877-022-03522-5)
Supplement: Supplementary file 1 — Additional file 1 Supplement Table 1. Standard measurement protocol of muscle quality index in NHANES. [file 12877_2022_3522_MOESM1_ESM.docx]

**Supplement table 1. Standard measurement protocol of muscle quality index in NHANES**

| low muscle strength | | |
| --- | --- | --- |
| Knee flexion/extension force | Male< 550.15 Newtons | Female<373.63 Newtons |
| low muscle quantity/quality | | |
| Appendicular skeletal muscle mass | Skeletal muscle index (SMI) less or equal to 7.0 | |
| low physical performance | | |
| Gait speed | Walking speed ≤0.8 m/s | |

1. The peak forces of knee extension were documented in Newtons and measured at one speed (60 degrees/second).
2. The muscle mass was measured by the dual-energy X-ray absorptiometry.
3. People’s gait speed was estimated on a 20 ft long test track at their habitual timed and pace.
